# Returning individual wearable sensor results to participants: perspectives on challenges and lessons learned

**DOI:** 10.3389/fdgth.2025.1569452

**Published:** 2025-03-25

**Authors:** Krista S. Leonard-Corzo, Shelby L. Bachman, Jennifer M. Blankenship, Ieuan Clay, Kate Lyden

**Affiliations:** VivoSense, Inc., Newport Coast, CA, United States

**Keywords:** return of results, patient centricity, research dissemination, transparency, wearable sensors

## Abstract

With increased adoption of digital health technologies in clinical trials, sponsors and investigators are often faced with the challenge of promoting participant compliance and engagement. One strategy that may provide value to participants and, as such, help improve compliance with digital health technologies is to return individual study results to participants. Clinical research participants have consistently expressed their desire to receive individual study results following participation in a trial, and trial sponsors and investigators are eager to comply. However, multiple challenges and barriers to its implementation mean that return of results is rarely carried out, despite alignment around its value. This perspective discusses the potential benefits of returning individual study results, including improved participant engagement and compliance, increased patient trust, and increased sense of health ownership. We also discuss the practical challenges of and barriers to returning individual study results from digital health technologies back to participants related to what, how, and when to return results. We assert that clinical trial sponsors and investigators should consider returning individual study results to participants and propose potential solutions to address specific challenges.

## 1 Introduction

With the growing capabilities of digital health technologies (DHTs), there has been increased integration of wearable and other sensors within clinical trials to measure a variety of outcomes (e.g., physical activity, sleep, eating habits, etc.) within a range of therapeutic areas (e.g., neurological conditions, cardiovascular conditions, etc.) (1, 2). Despite the increasing interest in and integration of wearable sensors in clinical trials, challenges to their successful integration continue to exist. For example, in the case of wearable sensors, participant wear compliance remains a major barrier that clinical trial sponsors and investigators face, as it can impact the quality, accuracy, and reliability of the data generated from these sensors (3, 4). This suggests the need for effective strategies to increase compliance. One potential patient-centric strategy is returning individual wearable sensor results to participants. Past research participants have consistently expressed their desire and expectation to receive a summary of their individual results after providing their time and effort in a clinical trial (5–10). As such, several organizations and funding agencies have called for the return of individual study results in response to participants' desires and to increase patient engagement and transparency (11–13). Although clinical trial sponsors and investigators agree that individual study results should be returned to participants (8, 14), questions persist regarding what, how, and when to return results. In this perspective, we discuss the benefits as well as the practical challenges of and barriers to returning individual study results from wearable sensors back to clinical trial participants. We discuss patient-centric approaches to returning individual study results derived from wearable sensors and propose potential strategies to address specific challenges.

## 2 Benefits of returning individual study results

With the emerging interest of returning individual study results, researchers have begun to ask research participants about their beliefs regarding the benefits of receiving individual study results. These studies are mostly hypothetical in nature (i.e., ask participant preferences without the actual return of results) and do not exclusively examine the benefits of receiving individual study results derived from DHTs specifically. However, given that these studies ask participants about preferences and expectations for receiving individual study results broadly, their findings can be used to inform the process of returning results derived from DHTs. Across several studies, participants have reported that receiving individual study results can help increase the sense of ownership of their data, facilitate discussions about their health with others, and help them to build health goals and drive healthy behavior change (6, 8, 14–16). Further, participants have reported that receiving individual study results, particularly if compared to some sort of benchmark (e.g., other similar individuals, their own data over time), can help motivate overall health goals (15). Participants have also reported that returning individual study results can improve participant recruitment and enrollment as well as participants' willingness to participate in future research studies (7, 8, 17). In addition, participants have reported that knowing they will receive individual study results encourages their participation in current trials and facilitates compliance (7). Specifically, receiving their results allows participants to feel more part of the research process and builds trust between themselves and researchers, which further encourages participation (7). Although these findings based on participants' reports are insightful, more research is needed to further understand these benefits and evaluate the actual impact of returning individual study results. For example, research examining whether returning individual study results increases compliance with study schedules and protocols, enrollment rates, and retention rates is warranted. Importantly, one study found that receiving individual study results did not lead to negative effects such as psychological distress, suggesting that the practice of returning individual study results can safely be implemented (18).

## 3 Challenges and potential strategies for returning individual study results

Although participants have indicated that the benefits of receiving study results outweigh any potential challenges (5), challenges do exist. In 2018, Wong and colleagues (19) published a viewpoint outlining the uncharted and untested questions of who, how, and when to return data to study participants. Since then, several studies have examined whether participants believe results should be shared, however continue to fall short in answering the specific questions of what, how, and when to return individual study results from wearable sensors specifically. This section will outline the challenges (Table 1) related to returning individual study results derived from wearable sensors that clinical trial sponsors and investigators may face as well as our perspective on potential strategies that may address these challenges.

**Table 1:** Summary of challenges of returning individual study results derived from wearable sensors and potential strategies.

Topic	Challenges
Delivering value to participants without creating undue burden (Section 3.1)	What data should be returned to participants?
	How would participants like to receive their individual results (e.g., mode of delivery)?
	How to summarize and contextualize individual results?
	How to ensure that results from wearable sensors are reliable and valid?
	What to return if data are missing and/or of poor quality?
Avoiding bias when returning individual study results (Section 3.2)	When should individual results be returned?
Balancing ethical considerations (Section 3.3)	Avoiding harm in clinical research.
	Support and/or explanation of individual results after delivery.
Operational execution (Section 3.4)	Who is responsible for returning individual study results to participants?
	What are the logistics behind returning individual study results?

### 3.1 Delivering value to participants without creating undue burden

Advancements in technology have enhanced the capabilities for wearable sensors to collect and measure real-world behavior within clinical trials. However, this has also resulted in an increase in the sheer volume of data that can be collected and derived. For example, one wearable sensor can capture data to derive a variety of metrics including number of steps, time spent walking, sedentary time, different intensities of physical activity, various sleep metrics, heart rate variability, and more. Not to mention, these metrics can be analyzed on different levels of granularity—daily, weekly, or monthly. Thus, the challenge becomes what data should be returned to participants. In 2017, the Multi-Regional Clinical Trials Center of Brigham and Women's Hospital and Harvard released recommendations outlining what types of results should be returned to participants (20). With respect to individual study results, they categorized the data into four groups: (1) Data Type A, urgent results and urgent incidental findings; (2) Data Type B, routine results and non-urgent incidental findings; (3) Data Type C, end of study individual results; and (4) Data Type D, exploratory results. They recommend that at minimum, Data Type A and Data Type C (study group and assignments and primary endpoints) should be returned to participants. It may be useful for investigators to categorize their data derived from wearable sensors as so to determine what to return to participants. Another potential solution is to prioritize returning the results that participants deem meaningful. This requires engaging with participants directly, either through quantitative and/or qualitative methods, to identify metrics they find relevant, actionable, and helpful for managing their health and/or behaviors. Investigators could consider applying a data driven approach (e.g., machine learning) to identify candidate meaningful metrics to return to participants by examining associations of relevant variables (e.g., predictors of behavior change) for different populations (21). However, investigators should still consider engaging with participants directly to confirm meaningfulness. This can occur before and/or after returning individual study results, which would allow investigators to refine the return of results process based on participant feedback. However, few studies have explored the feasibility and value of this. Sayeed and colleagues (15) found that participants preferred to receive results related to genetic data followed by heart imaging, study watch activity data, and microbiome data. While promising, more research is needed to understand what types of data from wearable sensors participants find most meaningful. Adopting an iterative approach that asks participants about their perspectives, and subsequently using their feedback to refine the return of results process, may foster continuous patient engagement and ensure the return of meaningful results.

Similarly, participants have provided some insight on how they think they would like to receive individual study results. Specifically, participants discussed that appropriate modes of delivery include email/text, paper mail, face-to-face communication, social media, and on websites (6–8, 17, 22, 23). Some evidence suggests that preferences for mode of delivery may differ based on personal characteristics. For example, younger participants tend to prefer email/text or online avenues whereas older participants prefer a hard copy received by mail or in-person (7, 8). Some may want an accompanying phone call or training, particularly if results include sensitive information that could unintentionally cause harm or distress (7, 23). Ultimately, participants emphasize the importance of personalization and believe that they should be given the choice of how they receive their individual study results (17, 22). Equally important is the challenge of summarizing and contextualizing results in a manner that participants find understandable and meaningful. We recently conducted a study where participants received individual study results derived from wearable sensors and obtained their feedback and opinions about their preferences for the quality and content of the received reports (24). Participants had overall positive feelings towards the report, however had feedback that would help improve the digestibility of the reports (e.g., including more contextual information, presenting the information differently, etc.). Past research has also shown that some participants prefer visuals such as graphs, whereas some participants prefer words or videos (17, 22). Tailoring reports to these preferences may enhance comprehension and engagement. Overall, investigators may want to consider how to best deliver and convey sufficient information without unnecessary complexity that fits the desires of participants. To best provide value back to participants, previous work suggests that researchers should offer participants multiple options on how to receive results, ask participants their preferences prior to returning their results, and then when results are returned, be available to explain the results to the participant and answer questions.

In addition to returning meaningful results that are summarized in a digestible manner, it is essential to ensure that the results derived from wearable sensors are reliable and valid. By using validated algorithms, investigators can ensure that they are returning accurate and reliable results to participants. Another challenge is what to return if data are missing and/or of poor quality. Despite strong efforts to maximize data collection, non-wear time is still a common issue clinical trial sponsors and investigators face with the implementation of wearable sensors. Returning individual results to participants summarized from data with missing cases due to non-wear may not provide them with an accurate representation of their data and behavior. Applying established, *a priori* decision rules to define valid days of data and only summarizing and returning metrics if the data meet these thresholds (e.g., for measurement of activity with actigraphy, typically 4 valid days including 1 weekend day with valid days defined as 10 h or more of wear time is applied) (25) may provide more accurate summarizations of participants' real-world behavior. Given that investigators may still want to continue to return individual study results as it may help increase compliance (7), as a middle ground, rather than not returning any results due to missing data, investigators may want to consider returning a report thanking participants for their time and summarizing the amount of data they contributed to the study (e.g., wear time). Nevertheless, transparency and clearly communicating to participants how their results were summarized if and/or when data are of low quality or missing is key.

### 3.2 Avoiding bias when returning individual study results

Clinical trial sponsors and investigators have voiced concerns surrounding the potential for introducing bias or unintended behavioral changes if participants receive their results before the trial has ended (i.e., Hawthorne effect) (26). Thus, returning results after a trial has concluded may be an approach that avoids introducing bias. However, the question of when to return results even after a trial has ended remains. For example, when returning results immediately after the trial may not be feasible (e.g., due to resource constraints), it is unclear as to whether participants will continue to find their results meaningful after long periods of time have passed. One approach may be to ask participants when they would like to receive their individual study results and if they would like to receive them closer to study completion. Although returning results at the end of a trial may sound most appealing, participants may also express a desire to receive their results throughout the trial, particularly with wearable sensors that can provide continuous data in near real-time. Therefore, there is a need to carefully weigh this desire against the potential risk of biasing the study. A potential middle-ground approach for studies where returning full results mid-trial is not feasible, is to provide participants with periodic updates on their compliance, to give them an ongoing sense of how much data they have contributed. This may help participants stay engaged with the study without receiving detailed behavioral data that could impact their actions and may also help maintain compliance, which is important in studies involving wearable sensors.

### 3.3 Balancing ethical considerations

Participants have expressed that they feel it would be unethical to withhold any information (7), thus the return of individual study results can be seen as an ethical imperative for researchers. Yet, this must be balanced against the need to avoid harm in clinical research. For example, some participants have voiced that they are concerned with the potential for their information to be stolen and/or used for marketing purposes (5). In addition, there may be negative emotional consequences for participants who receive unexpected results and/or misinterpret their results. A potential strategy may be to include information on the report describing how participants should interpret their results along with additional resources for participants. Moreover, reports may need to include a disclaimer that explains that the purpose of these reports are for research rather than “clinical care” and should not be used to make decisions about their health without guidance from healthcare providers. That is, although participants would like to receive information about actionable results to manage their health goals, it should be clarified that this should not be done without consulting a healthcare provider. Clinical trial sponsors and investigators may also want to consider including instructions for follow-up support (e.g., referral to healthcare providers, additional tests, counseling, etc.). Nevertheless, these ethical considerations highlight the possible value of providing participants with a choice of whether they want to receive individual study results, which is consistent with past research showing that participants have expressed that they should have a choice to opt out of receiving individual study results (22). Therefore, investigators could consider incorporating an opt in vs. opt out choice within the informed consent to give participants control over the return of individual study results process.

During the informed consent process, as well as during the delivery of results, it may also be valuable to provide participants with an explanation of the content of the returned results as well as any associated limitations. For example, there may be instances where the results on the provided reports may differ from results they see on their own consumer-grade wearable sensors. It is important to note that different wearable sensors apply different algorithms with varying degrees of accuracy when summarizing data. Subsequently, different wearable sensors may not provide the same results (e.g., consumer-grade wearable sensors may overestimate physical activity in real-world settings compared to research-grade sensors) (27, 28). Thus, when clinical trials return individual results derived from research-grade sensors, investigators may want to explain any potential differences participants may see in their reports compared to their own consumer-grade wearable sensors.

### 3.4 Operational execution

Despite its potential value, returning individual study results derived from wearable sensors to participants requires time and resources. One major challenge includes the question of who is responsible for returning individual study results to participants (i.e., does it fall on the study team, the sites, or the sponsor). Some researchers have suggested that this responsibility is likely with the lead investigator(s) and that developing a communication plan can help provide oversight (29). Nevertheless, the selected communicator should have the expertise and resources needed to communicate results with participants and address any potential concerns. Another challenge includes the logistics of returning individual study results to ensure it is done in an efficient manner while maintaining accuracy. The entire process of returning individual study results derived from wearable sensors includes processing and cleaning the wearable sensor data, identifying and summarizing relevant metrics, generating a report that incorporates relevant metrics into visualizations and/or text, and sending reports back to participants. This is often a time-consuming process, particularly in clinical trials with large sample sizes. However, in the case of wearable sensor data, relying solely on a fully automated process may not allow for data quality checks to ensure that reported metrics are reliable and accurate. Thus, a hybrid approach may be preferable wherein automation is leveraged for data processing and report generation, however critical oversight and quality assurance is performed by investigators. This approach balances efficiency with accuracy and ensures that participants receive reliable and meaningful results.

## 4 Discussion

The rise of digital health technologies, particularly wearable sensors, has allowed for the generation of large volumes of real-word participant data within clinical trials. The practice of returning individual study results derived from wearable sensors aligns with increasing calls for transparency and patient engagement in research. Moreover, it fulfills the desires of both participants and clinical trial sponsors and investigators. Returning individual study results can encourage participation in clinical trials, foster trust between participants and researchers, allow participants to feel more involved in the research process, and motivate healthy behavior change. Further, returning individual study results derived from wearable sensors may help with compliance, subsequently increasing the quality of the data collected.

While the benefits of returning individual study results derived from wearable sensors are compelling, the practical challenges of implementing this practice are real and cannot be overlooked. Specifically, challenges of what, how, and when to return individual study results as well as ethical challenges persist. Addressing these challenges is critical to ensuring the process is both participant-centric and feasible for clinical trial sponsors and investigators. Although current research attempting to address these challenges are promising, the majority of studies examining what, how, and when to return individual study results have been hypothetical and not focused on returning results derived from DHTs. Future studies that actively return individual study results to participants and obtain their feedback on the results and the implemented process are warranted. For example, this process can be embedded within larger existing studies to obtain participant feedback and subsequently refine the return of individual study results process. We recently conducted such a study, which will be one of the first to our knowledge that actively return individual study results derived from wearable sensors to participants and used their feedback to refine the process for future implementation (24). Nevertheless, this perspective introduced potential strategies that may help address challenges associated with returning individual study results derived from wearable sensors, however additional research is needed to determine whether these strategies are feasible. Examining the impact of these strategies may be worthwhile given that returning individual study results not only creates a transparent and empowering research process for participants, but can also unlock the potential to facilitate high quality research that incorporates DHTs and help advance patient-centered care and research.

## Data Availability

The original contributions presented in the study are included in the article/Supplementary Material, further inquiries can be directed to the corresponding author.
